# Doctor, what does my positive test mean? From Bayesian textbook tasks to personalized risk communication

**DOI:** 10.3389/fpsyg.2015.01327

**Published:** 2015-09-17

**Authors:** Gorka Navarrete, Rut Correia, Miroslav Sirota, Marie Juanchich, David Huepe

**Affiliations:** ^1^Psychology Department, Laboratory of Cognitive and Social Neuroscience, UDP-INECO Foundation Core on Neuroscience, Universidad Diego PortalesSantiago, Chile; ^2^Faculty of Education, Universidad Diego PortalesSantiago, Chile; ^3^Department of Psychology, Kingston UniversityKingston upon Thames, UK; ^4^Department of Management, Kingston UniversityKingston upon Thames, UK

**Keywords:** Bayesian reasoning, positive predictive value, risk communication, Bayesian textbook tasks, medical tests

## Abstract

Most of the research on Bayesian reasoning aims to answer theoretical questions about the extent to which people are able to update their beliefs according to Bayes' Theorem, about the evolutionary nature of Bayesian inference, or about the role of cognitive abilities in Bayesian inference. Few studies aim to answer practical, mainly health-related questions, such as, “What does it mean to have a positive test in a context of cancer screening?” or “What is the best way to communicate a medical test result so a patient will understand it?”. This type of research aims to translate empirical findings into effective ways of providing risk information. In addition, the applied research often adopts the paradigms and methods of the theoretically-motivated research. But sometimes it works the other way around, and the theoretical research borrows the importance of the practical question in the medical context. The study of Bayesian reasoning is relevant to risk communication in that, to be as useful as possible, applied research should employ specifically tailored methods and contexts specific to the recipients of the risk information. In this paper, we concentrate on the communication of the result of medical tests and outline the epidemiological and test parameters that affect the predictive power of a test—whether it is correct or not. Building on this, we draw up recommendations for better practice to convey the results of medical tests that could inform health policy makers (What are the drawbacks of mass screenings?), be used by health practitioners and, in turn, help patients to make better and more informed decisions.

## Introduction

Research in Bayesian reasoning started with the pioneering work of Casscells (1978) and Eddy (1982) and has consisted mostly in asking participants about the trustworthiness of positive results in screening tests, i.e., the positive predictive value (PPV) of medical tests. The PPV of a test expresses the proportion of people affected by a medical condition relative to the total number of positive test results. Textbook Bayesian problems (as well as medical tests' brochures, informed consent forms, etc.) commonly present information about the prevalence of a condition (i.e., proportion of population with the condition), the sensitivity of a test (i.e., probability that a test detects the presence of the medical condition) and its false-positive rate (i.e., probability that the test detects a medical condition that is not present), and ask participants to assess the positive predictive value of the test (PPV). The following example (Gigerenzer and Hoffrage, 1995) is a widely used Bayesian reasoning problem:

The probability of breast cancer is 1% for women aged forty who participate in routine screening. If a woman has breast cancer, the probability is 80% that she will get a positive mammogram. If a woman does not have breast cancer, the probability is 9.6% that she will also get a positive mammogram. A woman in this age group has a positive mammogram in a routine screening. What is the probability that she actually has breast cancer?

To answer the question of PPV correctly—the probability of having the medical condition given a positive test result, formalized as p(H|D)—participants need to understand the structure of the problem and extract the key probabilistic pieces of information outlined above: the prevalence of the condition [p(H) = 1%], and the test characteristics—sensitivity (p(D|H) = 80%) and false-positive rate (p(D|~H) = 9.6%).

In this example, to adequately answer the question (PPV), a participant (or a patient) would need to combine all the above information in a specific way, following the Bayes' formula as displayed in Equation (1).

(1) $$\begin{array}{l} p\left(H|D\right)=\frac{p\left(H\right)p\left(D|H\right)}{p\left(H\right)p\left(D|H\right)\;+\;p\left(\sim H\right)p\left(D|\sim H\right)} \end{array}$$

Bayesian problems vary in complexity depending on the format of presentation of the probabilistic information (e.g., natural frequencies vs. single-event probability) and based on the structure and content of the narrative (Barbey and Sloman, 2007; Krynski and Tenenbaum, 2007; Lesage et al., 2013; McNair and Feeney, 2014). There are ways to simplify the computational demands: using absolute reference class (e.g., frequencies or chances with a natural sampling) and specifying the number of positive tests p(D). In this case, with only two pieces of information, p(D&H)—the chances of having a positive result and the disease at the same time—and p(D)–the chances of a positive test—we can proceed using a simplified version of the Bayes' theorem outlined in Equation (1). Equation (2) could be seen as a simple case of Laplacian probability (Laplace, 1810): *ratio of “favored events” to total possible events* (i.e., ratio of the number of correct classifications to the total positive results in the test).

(2) $$\begin{array}{l} p\left(H|D\right)=\frac{p\left(D\;\&\;H\right)}{p\left(D\right)} \end{array}$$

Researchers have found that the ability of people to solve Bayesian problems depends greatly on the way the information is conveyed, ranging from ~5% in the first case (1), to up to ~50% in the latter (2) (see Gigerenzer and Hoffrage, 1995 for a very detailed explanation encompassing the difference between Equations 1 and 2). Manipulating features of the textbook Bayesian problems such as visual representations (Brase, 2009; Sirota et al., 2014b), clarification of the causal structure (Krynski and Tenenbaum, 2007; McNair and Feeney, 2014), and information structure (Barbey and Sloman, 2007) can also improve reasoning performance in some circumstances. Individual differences also account for some performance variance over and above the actual content of the task, such as, for example, cognitive reflection ability and numeracy (Sirota and Juanchich, 2011; Johnson and Tubau, 2013, 2015; Lesage et al., 2013; Sirota et al., 2014a).

Furthermore, the way we currently study Bayesian reasoning may not be the best. It has been argued that research focused on how people update their beliefs or probabilities, to improve our knowledge about how the mind works, assesses ability more akin to statistical inference than to Bayesian reasoning (Mandel, 2014). But, more specifically, if we are interested in the best way to convey medical information to patients, we need to adopt a more flexible approach than the mechanical application of textbook problems. Indeed, most of the research outlined above used textbook problems to study the theoretical basis of Bayesian reasoning (Baratgin and Politzer, 2006), often using the presence of this type of information in medical contexts as a testimony of the importance of the research. The focus has been on ways to improve people's understanding via the use of pictorial aids, causal structure, computational simplification, clarification of the structure of the problem and boundary conditions (e.g., individual differences in cognitive processing), sometimes forgetting the real needs of the applied side of our research.

The importance of finding better ways to communicate medical risks has become a common motivating factor for a fair share of the Bayesian reasoning literature, given the real world impact of this field and the fact that only a few people can actually understand this kind of information as it is commonly presented (see Sedlmeier and Gigerenzer, 2001; Juslin et al., 2011; Pighin et al., 2015a). Even health-care professionals often have difficulties understanding probabilistic information^1^ (Ghosh et al., 2004; Gigerenzer et al., 2007). Bayesian reasoning research has shown that people's understanding of probabilistic problems depends on the complexity of the structure of the problem, the computation required and their own cognitive skills and thinking styles. However, those principles rarely transcend the basic research walls. In clinical practice, what we know about Bayesian reasoning is not generally applied to improve the way of communicating risk. As a consequence, people have to understand their health practitioners' explanations, “informed consent” or medical tests brochures, where the information given is poorly structured, incomplete and simply often beyond their capabilities. The example below^2^ shows a prenatal test brochure for Down Syndrome. As far as we have seen, this is fairly representative of the prenatal tests' brochures available online. The explanation provided in the brochure is a mix of frequencies and relative probabilities from which it is very difficult to derive the positive predictive value of the test.

*It is estimated that trisomy 21 is present in 1 out of every 800 births in Canada*.

*It is estimated that trisomy 18 is present in approximately 1 out of every 6,000 births*.

*It is estimated that trisomy 13 is present in approximately 1 out of every 16,000 newborns*.

The Harmony Test has been shown to have detection rates of up to 99 % and false positive rates as low as 0.1 % for trisomy 21, 18, and 13 (…)

In this example, if a couple expecting a baby wanted to understand what a positive result in the test meant, they would have to deal with a very complex calculation. The information given can be matched to Equation (1)—assuming you know that, p(~H) = 1-p(H). For the trisomy 21 case it would translate into Equation (3):

(3) $$\begin{array}{l} \;p(Trisomy\;21\;|+test)=\\ (A)\;\frac{1\;out\;of\;800\;\times \;99\%}{\left(1\;out\;of\;800\times 99\%\right)+\left(799\;out\;of\;800\times 0.1\%\right)}=\\ \;(B)\;\frac{0.123}{0.123+0.0998}=0.55 \end{array}$$

If the parents completed Equation (3)^3^ they would realize the probability of having a child affected with a trisomy 21, 18, or 13 given a positive test result, is, respectively, 55%, 14% and 6% (see Navarrete et al., 2014 for a more detailed account), likely to be below their expectations, given a generally shared high regard for medical tests (Gigerenzer et al., 2009).

In a medical context, it is important that people understand the risks, the pros and cons of undertaking a test and how to interpret the result afterwards. The role of the medical personnel is vital and, although the ethical dimension and other issues involved are beyond the scope of this article, we want to recognize their complexity. In any case, we could probably agree that it is important that patients are given the possibility of reaching a sufficient level of understanding to give a truly informed consent. Why then are we forcing participants and patients to deal with a non-trivial set of information, and then to perform a calculation generally too difficult for them? In most cases this translates into patients or doctors being unable to provide an informed consent and to blindly trusting medical tests or falling prey to bogus medical tests, and in uninformed politicians implementing policies promoting mass screenings for low prevalence diseases, where the positive predictive value is also low (e.g., as for the Trisomy 13 for which a positive test identifies correctly the Syndrome in only 6 cases out of 100). This can result in negative consequences, costing life and money (Gigerenzer et al., 2007).

But why are mass screenings less useful than targeted screenings? To be able to understand the result of a medical test, one needs to take into account two different and inter-related sets of information. The first set of information relies on the test's characteristics: its sensitivity and false positive rate. The second set of information has to do with the disease itself, more specifically its prevalence. The usefulness and trustworthiness of a test critically depends on the prevalence of the medical condition it is seeking to detect, and this depends on the reference group used (Baldessarini et al., 1983).

Prevalence,– and its relationship with false positives– is pivotal and very often misunderstood when interpreting the meaning of a positive result in a test. As prevalence decreases—as is the case in mass screenings—even near perfect tests produce a large number of false positives, and hence, a low PPV. Several authors have warned about the dangers of mass screenings and their negative consequences, such as the high cost of false positives in psychological and monetary terms (Christiansen et al., 2000; Gigerenzer et al., 2007; Navarrete et al., 2014).

It is important to keep in mind that prevalence is not a characteristic of a test but of the population to whom the test is given. For example, the prevalence of certain chromosomal aberrations in fetuses is related to maternal age and gestation time (Nicolaides, 2004). The exact same test would “work” a lot better—i.e., have a higher PPV—in older pregnant women than in younger ones. Specifically, the rates of prevalence range from 1 out of 1000 for 20 year old mothers up to 1 in 38 for 42 year old mothers (Nicolaides, 2004, p. 18). That means that the combined test reliability, used commonly as a screening procedure, goes from a 2% PPV when used in young mothers to 34% PPV when used in a relatively high risk group. Still a far cry from a reliable test, but a change with dramatic consequences given the default recommended assessment in the case of a positive result, and its associated risks (Navarrete et al., 2014).

To combine all available information, one should follow Equation (2): ratio of the number of correct classifications to the total positive results in the test. The number of correct classifications will always be close to 1 as the prevalence is usually presented in a standard way—*1* out of X (but see Pighin et al., 2015b for some related issues)—and the sensitivity is usually close enough to 100%. On the other hand, the denominator magnitude will depend on the number of false positives and the *X* term of the prevalence (1 out of *X*). Imagine we have a test with a 0.1% rate of false positives that aims to detect a relatively common condition affecting 1 in 100 individuals (see Equation 4). The number of false positives would be calculated multiplying the 99 healthy individuals by 0.1%, that is, 99 × 0.001 = ~ 0.099. Using Equation (2), this would translate into a PPV of 0.91, or a 91% chance of having the medical condition given a positive test result.

(4) $$\begin{array}{r} p\left(H|D\right)=\frac{p\left(D\&H\right)}{p\left(D\right)}=\frac{1}{1+0.099}=0.91 \end{array}$$

Unfortunately, tests are not always so reliable, nor are the tested medical conditions so common. According to the EU regulations^4^, most patients suffer from diseases affecting 1 in 100,000. Test reliability and prevalence can dramatically reduce the ability of a test to identify a medical condition. For example, a test with the same rate of false positives (0.1%) that aims to detect a disease with a lower incidence, such as of 1 in 10,000 would result in a much lower PPV: 0.09, or 9%, as seen in Equation (5).

(5) $$\begin{array}{r} p\left(H|D\right)=\frac{p\left(D\&H\right)}{p\left(D\right)}=\frac{1}{1+9.99}=0.09 \end{array}$$

The previous two examples show how the PPV of a test can change from 91 to 9% simply because of a lower incidence of a medical condition (from 1 in 100 to 1 in 10,000). In a mass screening campaign, the incidence of a medical condition is lower than in a targeted screening campaign, lowering dramatically the reliability of the test results.

Of course, as often happens, if a medical test is not as reliable as the one used in the two examples above (100% sensitivity, and 0.1% false positive rate), a low positive predictive value appears even with common medical conditions. For example, see in Equation (6) the computation of the positive predictive value of a test aiming to detect a condition with a prevalence of 1 in 100, and a false positive rate as low as 1%. When the rate of false positives increases by 0.9%, the positive predictive value of the test decreases by 40%, dropping from 90 to 50%. In this context, a person receiving a positive test has only a 50% chance of actually having the condition.

(6) $$\begin{array}{r} p\left(H|D\right)=\frac{p\left(D\&H\right)}{p\left(D\right)}=\frac{1}{1+0.99}=0.5 \end{array}$$

**Figure 1:**
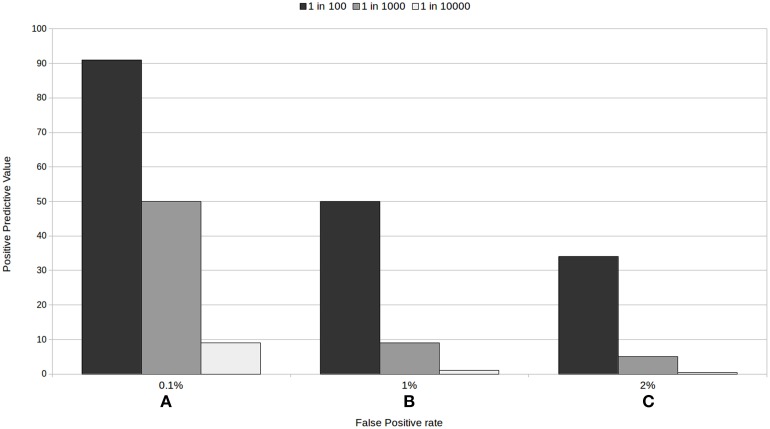
**Positive predictive value for three tests with a 100% sensitivity according to the rate of false positive (A) 0.1%, (B) 1%, and (C) 2%, and to the prevalence of the condition**.

With all these examples, we are not implying that screening tests should not be trusted. We intend to outline the factors needed to be considered when using and interpreting medical test results. As we have seen, low prevalence rates, and their interaction with false positive rates, are generally guilty of decreasing the positive predictive value of a test: Figure 1 provides an illustration of this. The variability of positive predictive values of medical tests, according to the characteristics of the test and the prevalence of the condition, makes it hard for patients to decide whether to take the test and to assess their chances of having a condition when they test positive, particularly when the information given to them is generally too complicated to understand.

Given the need of facilitating the patient's assessment and decision making powers, different solutions can be offered. Further medical research to improve the present tests and decrease their false positive rates is obviously a very important and necessary path. Testing only people in higher risk groups and avoiding mass screenings as much as possible or, at least, making their limitations clear, is a critical necessity given the reality of the medical tests available and their trustworthiness for diagnosing rare conditions. Of course, increasing public health literacy should be traversal to these and any other alternatives available (Gigerenzer, 2015).

Nonetheless, one important aspect not covered in the above options is that we need to find better ways to communicate medical risks, starting with using the information obtained through empirical research in medical practice. For those of us interested in improving the way we convey medical risks, focusing research on what real patients need is vital. In the real world, when receiving medical test results or reading informed consents, people are confronted with probabilistic information generally too complex to be understood, let alone calculated. We need to avoid altogether the classical triad (specificity, false positive rate and prevalence) if we want to improve people's chances of understanding test results and informed consents, and of playing a more active role in shared decision making. It is also important to acknowledge that there exist teams focusing on helping health practitioners better communicate risk and patients better understand risks (e.g., Reyna et al., 2009; Garcia-Retamero et al., 2010; Gigerenzer, 2014). However, theoretical research seems to still have the lion's share in Bayesian reasoning and we would suggest further harnessing these teams' work to derive simple and effective guidelines to communicate medical test results.

Our proposal, then, is to present information about the PPV, and specifically, how trustworthy a positive or a negative result in each particular test really is *for the individual*: that is, the PPV for the test relative to the risk group the person belongs to. Using epidemiological factors (such as age in the prenatal screening example above, a list of common behaviors for each risk group, family history, etc.) we could help people assign themselves to a specific risk group. An example would be to present something akin to one of the sections of Figures 1A–C, making clear which epidemiological factors, risk behaviors, etc. are associated with each of the prevalence or risk groups. In prenatal screening, this would depend, amongst other factors, on the age of the mother to be. In a mass screening context, this approach could translate to most people (low risk people) avoiding getting tested for rare conditions, as the PPV for them would be extremely low. Prevalence is a characteristic of the disease or of the group tested and its risk factors, and not of the test, and we must stop ignoring this fact. This would help people distinguish between good and bad tests and make for more informed decisions.

To sum up, the goal of this article is to call on the scientific community studying Bayesian reasoning to join efforts and focus further on finding better ways to present medical information. Such research could inform policy makers' decisions (specifically helping them understand why mass screenings are less useful than targeted screenings) and be used by health staff to enable patients to make better informed decisions related to their health. One possibility is to find good ways to assign people to risk groups and to present information about tests relative to these risk groups, but other options surely exist. Of course, it is important to empirically confirm that people really do better with this new way of presenting the information (e.g., they do understand the pros and cons of the combination of tests suggested in prenatal screening), and to assess the medical consequences of such trials. This call for further applied research is not unique and joins other initiatives to avoid risk miscommunication (e.g., fact-box for breast cancer screening pamphlets as suggested by Gigerenzer, 2014). Most people would agree: *misinformation needs to stop*. We have the chance to work toward this goal together.

### Conflict of interest statement

The authors declare that the research was conducted in the absence of any commercial or financial relationships that could be construed as a potential conflict of interest.

## Footnotes

^1^For example, Gigerenzer et al. (2007) show that the number of physicians able to solve a multiple choice breast cancer screening problem was 21%, slightly below chance.

^2^From http://www.prenatest.ca/en/Harmony-Prenatal-Test-Brochure.pdf.

^3^To be able to give a reference point, we asked 66 people to solve the above two Equations 3(A) and 3(B) through the web platform Amazon's Mechanical Turk, and the average accuracy correct response was 21 and 53%, respectively. That is, even when we give people the data of the brochure within the required formula, less than 25% are able to correctly solve it.

^4^From http://ec.europa.eu/health/rare_diseases/policy/index_en.htm: “In EU countries, any disease affecting fewer than five people in 10,000 is considered rare. That number may seem small, but it translates into approximately 246,000 people throughout the EU's 28 member countries. Most patients suffer from even rarer diseases affecting one person in 100,000 or more. It is estimated that today in the EU, 5-8,000 distinct rare diseases affect 6–8% of the population—between 27 and 36 million people.” The PPV for a test with 100% sensitivity and a 0.1% false positive rate trying to detect a 1 in 2000 condition is 50%.
